# Biomechanical Diversity of Mating Structures among Harvestmen Species Is Consistent with a Spectrum of Precopulatory Strategies

**DOI:** 10.1371/journal.pone.0137181

**Published:** 2015-09-09

**Authors:** Mercedes Burns, Jeffrey W. Shultz

**Affiliations:** BEES Program and Department of Entomology, University of Maryland, College Park, Maryland, United States of America; Scientific Research Centre, Slovenian Academy of Sciences and Arts, SLOVENIA

## Abstract

Diversity in reproductive structures is frequently explained by selection acting at individual to generational timescales, but interspecific differences predicted by such models (e.g., female choice or sexual conflict) are often untestable in a phylogenetic framework. An alternative approach focuses on clade- or function-specific hypotheses that predict evolutionary patterns in terms neutral to specific modes of sexual selection. Here we test a hypothesis that diversity of reproductive structures in leiobunine harvestmen (daddy longlegs) of eastern North America reflects two sexually coevolved but non-overlapping precopulatory strategies, a primitive solicitous strategy (females enticed by penis-associated nuptial gifts), and a multiply derived antagonistic strategy (penis exerts mechanical force against armature of the female pregenital opening). Predictions of sexual coevolution and fidelity to precopulatory categories were tested using 10 continuously varying functional traits from 28 species. Multivariate analyses corroborated sexual coevolution but failed to partition species by precopulatory strategy, with multiple methods placing species along a spectrum of mechanical antagonistic potential. These findings suggest that precopulatory features within species reflect different co-occurring levels of solicitation and antagonism, and that gradualistic evolutionary pathways exist between extreme strategies. The ability to quantify antagonistic potential of precopulatory structures invites comparison with ecological variables that may promote evolutionary shifts in precopulatory strategies.

## Introduction

Understanding the rapidly-evolving and species-specific morphologies of genitalia and other sexually dimorphic structures is a long-standing goal of evolutionary biology [1–3], with recent efforts focusing on how female mate choice and sexual conflict operate at individual or generational timescales within populations or species [4–6]. However, attempts to explain the origin and expansion of interspecific diversity by extrapolating such models to phylogenetic timescales is problematic given persistent debate about basic concepts (such as the relationships between female mate choice and sexual conflict) and the often non-exclusive predictions of such models [7–9]. An alternative perspective is to test clade-specific and function-based hypotheses that predict interspecific patterns in reproductive morphology, such as the order, direction, and co-occurrence of evolutionary change in homologous reproductive behaviors and structures [10–15] that do not rely on specific models of sexual selection. Here we use interspecific comparisons of reproductive morphology in the eastern North American clade of leiobunine harvestmen (daddy longlegs) [16–17] to explore evolutionary change in two precopulatory strategies: solicitous courtship via nuptial gifts, and precopulatory antagonism via mechanical forces [15] (where "antagonism" refers to apparent conflict during behavioral interactions rather than to differential fitness between contestants or specific microevolutionary processes).

**Fig 1:**
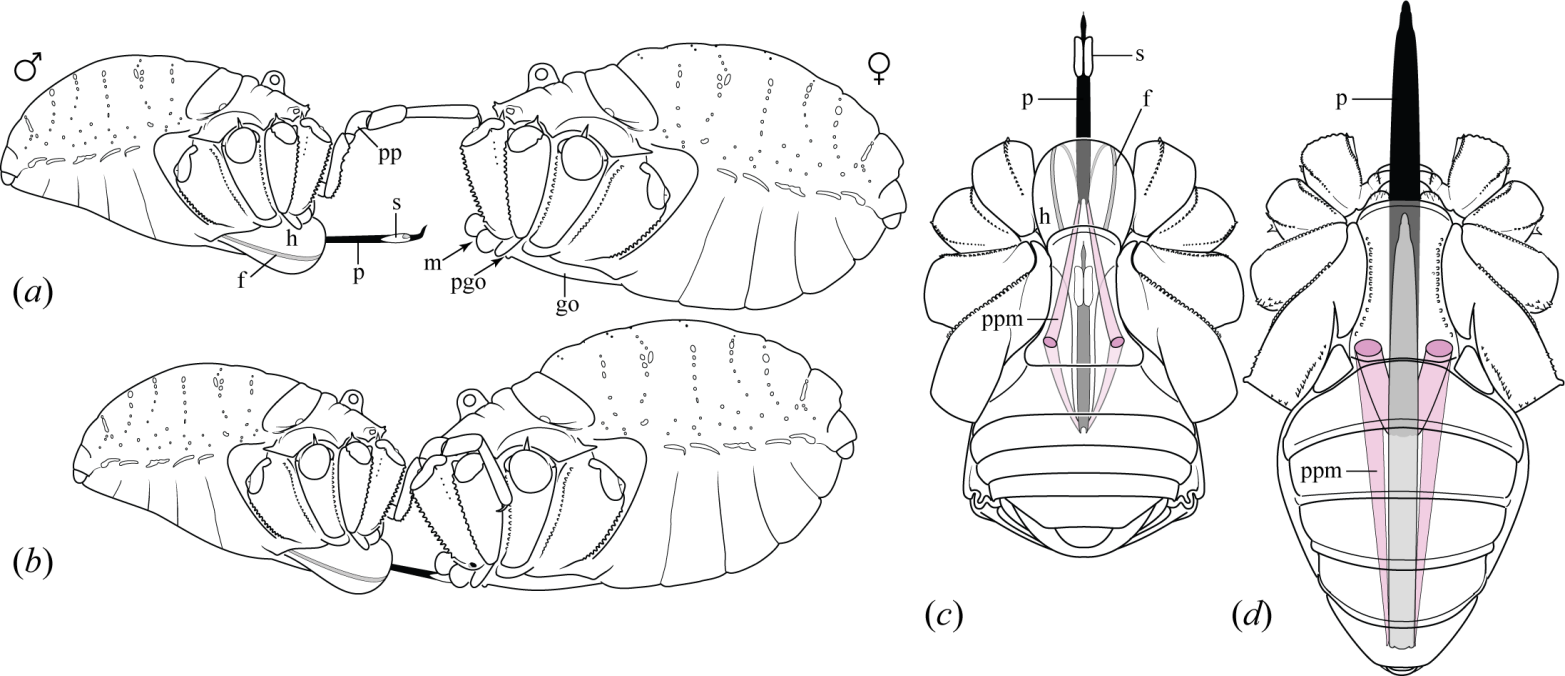
Summary of mating and male morphology in leiobunine harvestmen. (*a*) Precontact between male and female *Leiobunum verrucosum* (male left, female right), with legs removed for clarity. (*b*) Precopulatory position of mating pair. Male pedipalps grasp female behind coxae of second pair of legs; penis is inserted into female's mouth and then positioned at pregenital opening. (*c*) Ventral view of male *L*. *verrucosum* with short penis retracted and extended by hydraulic expansion of hematodocha. (*d*) Ventral view of male *Hadrobunus fusiformis* (not included in study but anatomically similar to *L*. *hoffmani*) with long penis retracted and extended by protractor muscles. Abbreviations: f, fultura; go, genital operculum; h, hematodocha; m, mouth; p, penis; pgo, pregenital opening; pp, male pedipalp; ppm, penis protractor muscle; s, nuptial gift sac.

Detailed studies of mating in leiobunine harvestmen (Fig 1*A* and 1*B*) began very recently [5,18,19] and fundamental insights continue to emerge. Notably, field observations once suggested that precopulatory behavior is simple or effectively absent, with copulation or rejection occurring rapidly upon incidental physical contact [20–23]. Yet video-based observations revealed a variety of close-contact precopulatory behaviors that superficially resemble copulation. Previous misinterpretations can be attributed to precopulatory interaction of the penis with the anatomically adjacent oral and pregenital openings of the female (Fig 1*A* and 1*B*). This can include the rapid delivery of nuptial gifts to the female's mouth via penile sacs and persistent contact between the penis and the pregenital opening, including apparent forceful penetration in some species [18,19]. Mating behavior is still unknown for most species, and inferences about precopulatory strategies have tended to rely on comparative functional morphology [5].

**Fig 2:**
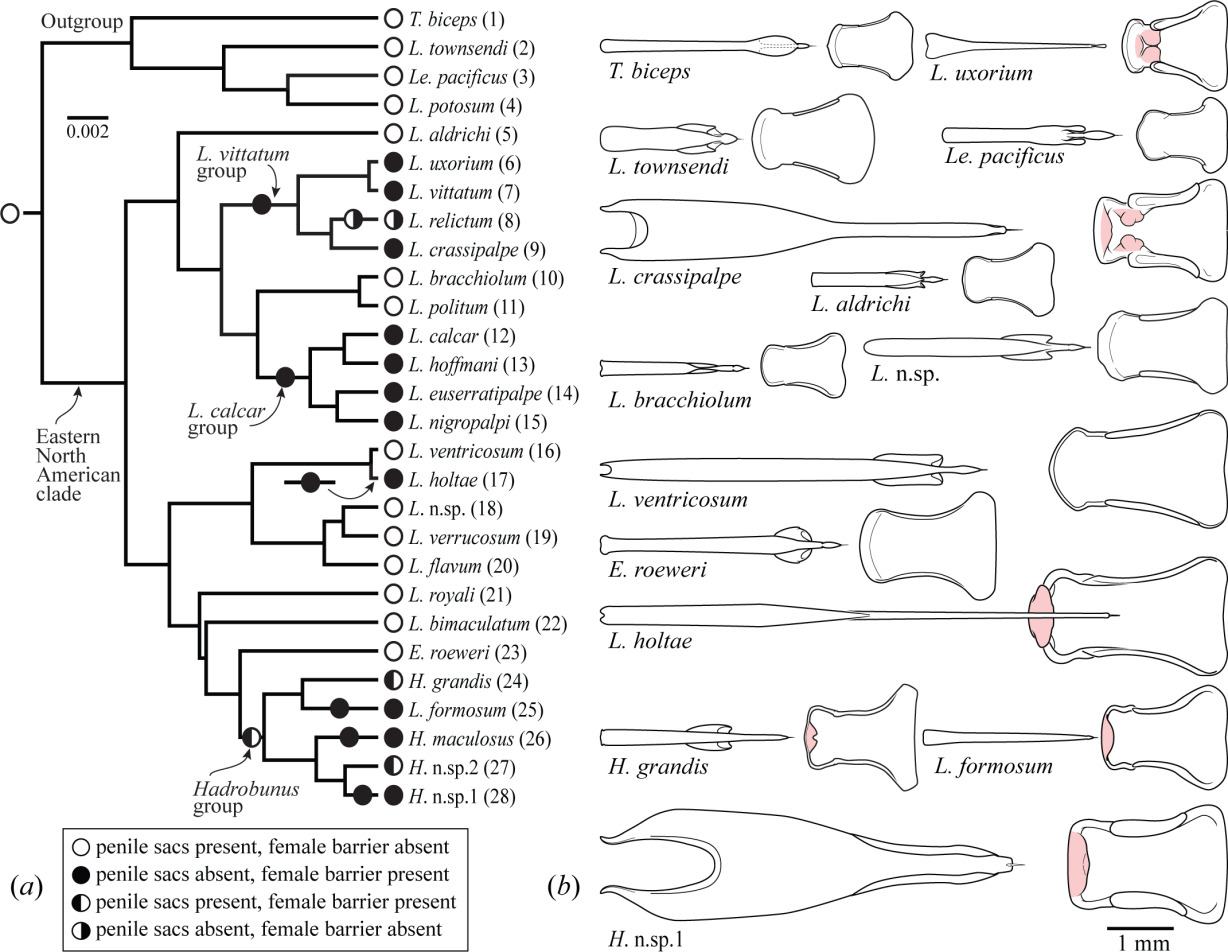
Phylogeny of leiobunines of eastern North America and examples of genitalic diversity. (*a*) Maximum clade credibility tree of 28 species used in phylogenetic comparative analyses, with parsimonious distribution of character-state combinations of penile nuptial gift sacs and female pregenital barrier mapped on branches. Scale = substitutions per site. (*b*) Examples of penes (dorsal view) and female genital operculum (internal dorsal view), with sclerotized pregenital barrier shaded. All drawings to same scale. Genus abbreviations: *E*, *Eumesosoma*; *H*, *Hadrobunus*; *L*, *Leiobunum*; *Le*, *Leuronychus*; *T*, *Togwoteeus*. Numbers after species names are used as data-point labels in Fig 3.

We recently surveyed diversity in the reproductive morphology in leiobunine harvestmen [15] and examined the phylogenetic distribution of two binary traits: presence or absence of subterminal penile sacs that deliver secreted nuptial gifts (Figs 1*A*–1*C* and 2*B*), and presence or absence of sclerotized pregenital armature in females (Fig 2*B*, S1 Fig). Of the four possible male/female-trait combinations, two were widespread among the 29 species examined, one combination being consistent with solicitous courtship (males with gift-bearing sacs, females without pregenital barriers), and the other with precopulatory antagonism (males without sacs, females with pregenital barriers). The two remaining combinations occurred in just three species. Phylogenetic comparative analysis showed that the solicitous combination is primitive and was replaced at least five times by an antagonistic combination, with the loss of penile sacs and gain of female pregenital barriers being highly correlated and usually occurring on the same phylogenetic branch (Fig 2*A*). We also found a strong association between the loss of penile sacs and the gain of enhanced clasping pedipalps in males. Based on these findings, we hypothesized that sexual coevolution in leiobunine harvestmen had produced two morphological syndromes that reflect two general non-overlapping strategies: solicitous courtship via penis-associated nuptial gifts and precopulatory antagonism via mechanical force exerted by the penis against the female pregenital opening.

Here we used multiple continuous variables derived from mechanical functions of reproductive structures in both sexes to test our predictions that structural diversity has coevolved in the two sexes to produce two functionally-distinct precopulatory syndromes. We measured 10 functional variables with magnitudes that should vary in proportion to the intensity of mechanical force used in precopulatory interaction, i.e., the relative force that a structure can generate, transmit, or resist. These variables included cuticular investment in reproductive structures, the relative forces produced by three skeletomuscular lever systems, and estimates of penile flexural resistance. In keeping with our previous study, we found convincing evidence for sexual coevolution in structural traits. However, interspecific variation in structure was distributed within a solicitous-to- antagonistic spectrum rather than in separate clusters. These findings suggest that the sexes have coevolved to produce precopulatory strategies that differ in the intensities of both solicitation and mechanical antagonism. The ability to quantify interspecific patterns in antagonistic potential invites a search for correlations with ecological or life-history factors that may explain the evolution of mating systems at phylogenetic time scales

## Methods

### (a) Taxon sample and phylogeny

We collected specimens from 28 out of 29 described and/or non-problematic eastern North American leiobunine species [17] for inclusion in this project (due to a lack of replication, *Hadrobunus* n. sp. 2 was not included). To ensure the validity of phylogenetic comparative methods, specimens (119 male, 147 female) were collected from localities near those from which specimens used in previous molecular phylogenetic analysis were obtained [17] (S1 Table). They were preserved in 70–100% ethanol. To provide an evolutionary framework and to correct for variance due to shared evolutionary history, we based all comparative methods on a maximum clade credibility tree (Fig 2*A*) developed from a posterior distribution of trees reconstructed from nuclear and mitochondrial sequences [15,17].

### (b) Data

We used 10 structural variables—two from females and eight from males—with values that were predicted to increase in proportion to the mechanical forces employed during precopulatory antagonism (see Table 1 for summary). Measurements (in mm) were made in ImageJ v. 1.44p [24] from photos obtained with a PaxCam digital camera mounted on either a Leica MZ APO dissecting microscope (0.63X or 1.0X objective lens, 8–80X zoom) or Wild Heerbrugg Makrozoom 1:5 with 6.2–32X zoom. Measurements were size-corrected (details below) and log-transformed to account for interspecific heteroscedasticity. Species means for each variable were obtained from three to 10 specimens (S1 Table).

**Table 1:** Overview of structural traits measured, including sex investigated, transformation of carapace width (*W*) for dimensional size-correction based on isometric expectation, and hypothesized effect on reproductive strategy.

Trait	Summary	Sex Investigated	Dimensional Size Correction	Hypothesis
**Female pregenital closing force**	**Estimated relative force of opercular levator muscle (See also S2 Fig)**	**Female**	**W2**	**Greater closing force with intensification of antagonism**
**Intrinsic penis muscle force**	**Estimated relative force of glans flexing muscle (See also S2 Fig)**	**Male**	**W2**	**Greater flexion force for antagonistic mating**
**Penis protractor muscle force**	**Estimated relative force of penile protractor muscle (See also S2 Fig)**	**Male**	**W2**	**Greater protraction force for resistance/stabilization in antagonistic mating**
**Penis length**	**Base to glans length of penis**	**Male**	**W**	**Longer penes indicate greater resistance to opposing forces, increases with antagonism**
**Penis investment**	**Mass of penis / body mass**	**Male**	**—**	**Cuticularization increases force transmission/ resistance during antagonistic mating**
**Male pedipalpal investment**	**Mass of pedipalps / body mass**	**Male**	**—**	**Cuticularization increases force transmission/ resistance during antagonistic mating**
**Female operculum investment**	**Mass of operculum / body mass (See also S3 Fig)**	**Female**	**—**	**Cuticularization increases force transmission/ resistance during antagonistic mating**
**Penis section modulus (SX)**	**Relative elastic strength in X-axis (See also S3 Fig)**	**Male**	**W4/W**	**Larger section moduli = greater elastic strength in antagonistic interactions**
**Penis section modulus (SY)**	**Relative elastic strength in Y-axis (See also S3 Fig)**	**Male**	**W4/W**	**Larger section moduli = greater elastic strength in antagonistic interactions**
**Fultura width**	**Width of longitudinal penile sclerites**	**Male**	**W**	**Wider fulturae store and return greater mechanical energy for antagonistic mating**

#### Correction for size effects

Body size was represented by carapace width measured at the junction of leg coxae I and II, a measurement that is largely unaffected by nutritional, reproductive, or preservational condition. Because relative values of many mechanical variables change predictably in systems that scale isometrically, we controlled for size by dividing each variable by the dimensionally appropriate transformation of carapace width (e.g., a mechanical variable that is expected to change in proportion to cross-sectional area was divided by the square of carapace width) (Table 1). Effectiveness of dimensional size correction was evaluated in two ways. First, we conducted a phylogenetic principal components analysis (pPCA) [25,26] on all variables, including male and female body size. Because the first principal component (PC1) is generally regarded as a size axis in morphometric analyses [27], exclusive or primary loading of body size on PC1 would suggest inadequate size correction by the dimensional method. Second, we conducted two sets of phylogenetic regressions between male and female variables, one using a dimensional size correction, and one using a phylogenetic approach (25,26). Differences in the statistical significance of correlations due to size correction methods would indicate a problem in dimensional size correction.

#### Relative female pregenital closing force

From basic lever mechanics, the closing force of the female genital operculum is the product of the force generated by the closing muscles (muscle 13 in [28]) and their mechanical advantage at the pregenital opening. The relative value for muscle force was inferred from the effective cross-sectional area of the muscle. Mechanical advantage was inferred from opercular anatomy (S2*A*–S2*C* Fig). Given that closing force should vary in proportion to effective cross-sectional area of the opercular closing muscles, this variable was size corrected by dividing it by the square of carapace width.

#### Relative forces of penis muscles

We estimated the relative forces generated by two penis muscles. The fibers of the intrinsic penis muscle (muscle 104 in [28]) (S2*D Fig*) arise from the walls of the penis and insert along a tendon that flexes the glans-shaft joint. The penis protractor muscle (muscle 101 in [28]) (Fig 1*C* and 1*D*) arises on the ventrolateral surface of the genital operculum and sternite, and inserts at the base of the penis (Fig 1*C* and 1*D*).

We randomly selected 3–6 fibers in each muscle and measured their cross-sectional areas and angles with respect to the long axis of the penis (intrinsic muscle) or body (protractor muscle). The relative force of each muscle was calculated by multiplying the average fiber cross-sectional area, fiber number and the cosine of the average fiber angle. Mechanical advantage was determined by measuring the input and output levers in ImageJ and multiplying input lever length by the relative muscle force to yield the total relative force divided by output lever length. We anticipated higher relative muscle forces in species with greater potential for precopulatory antagonism. Size effects on muscle-force estimates were corrected by dividing by the square of carapace width.

#### Penis length

This variable is related, in part, to the mechanics of penile protraction. Short penes are pushed forward by haemolymph pressure that everts the walls of the pregenital chamber; the exposed penis is thus mounted on a flexible, fluid-filled “balloon” (haematodocha) which offers low resistance to opposing forces (Fig 1*C*). In contrast, long penes are pushed forward throughout their excursion and resist opposing forces through contraction of the protractor muscles (Fig 1*D*). We thus anticipated relatively longer penes in species with greater intensities of precopulatory antagonism. Penis length was corrected for body size by dividing by carapace width.

#### Cuticular investment

The maximum mechanical force that a sclerite can transmit or resist likely varies in proportion to the amount of its constituent cuticle. We measured cuticular investment in three structures—the penis, male pedipalps and female genital operculum (Fig 1*A*). Each structure was removed from sample individuals. The body (minus legs removed at the coxa-trochanter joint) and the isolated structures were macerated in a 5% KOH solution at 65–68°C for 24–48 hours, rinsed in 100% ethanol, and dried overnight at 65–68°C. The mass of the body and each removed structure was determined with a Mettler Toledo MT5 microbalance (resolution 0.001 μg), and the ratio of the mass of each part to total body mass was calculated. Mass ratios required no size correction.

#### Section modulus of penis shaft

Harvestman penes can be modeled as hollow beams. When comparing a series of beams of similar composition, the relative magnitudes of several mechanical parameters can be estimated from cross-sectional profiles [29]. For example, a beam's flexural stiffness increases with both the amount of material that resists bending and its distance (*d* ^4^) from the beam’s flexural axis, where dorsal or ventral bending has a horizontal (*X*) axis and lateral bending has a vertical (*Y*) axis (S3 Fig). Flexural stiffness is estimated by the second moment of area (*I*_*X*_, *l*_*Y*_). We compared penes using elastic section moduli (*S*_*X*_, *S*_*Y*_), which are calculated as

$$S_{X}{=I}_{X}{/d}_{Y}{}_{max}$$

$$S_{Y}{=I}_{Y}{/d}_{X}{}_{max}$$

Elastic section modulus estimates a beam’s relative elastic strength (i.e., the smallest flexural force that will permanently damage the beam). Because the highest tensile and compressive forces experienced in bending occur farthest from the flexural axis, the material located at the maximum radius (*d* max) will be the first to fail. We anticipated larger section moduli in species that experience greater intensities of precopulatory antagonism.

Penes were isolated and embedded in JB-4 plastic medium (Electron Microscopy Sciences) following manufacturer’s specifications. Samples were oriented longitudinally in polymerization blocks following [30] and maintained overnight at 4°C under vacuum. Two to three 5-μm sections were obtained from the mid-shaft using a Microm HM 325 microtome. Outlines of the cuticle and lumen of each section were traced from digitized photographs using a Wacom Pen Tablet in Adobe Photoshop CS4 to create high-contrast images (S3 Fig). Images were imported into ImageJ and values for *S*_*X*_ and *S*_*Y*_ were obtained using MomentMacroJ [31]. Each value was corrected for size by dividing by the cube of carapace width.

#### Penile fulturae

The flexible walls of the male pregenital chamber contain a bilateral, ventrolateral pair of longitudinal sclerites that articulate posteriorly with the base of the penis and anteriorly with the anterior margin of the genital operculum (Fig 1*A*–1*C*). The sclerites bend during protraction and appear to act as springs that assist or stabilize penis movement. We predicted that wider fulturae store and return more mechanical energy than narrower fulturae and thus that wider fulturae are more likely to be associated with precopulatory antagonism. We corrected for size by dividing fultural width by carapace width.

### (c) Data analysis

Species means were compiled and imported into the R software environment [32]. We estimated phylogenetic signal in the data using Pagel's lambda [33,34] calculated in the *geiger* package [35]. Although lambda was found to be very low for all variables, we used appropriate comparative tests to compensate for any residual phylogenetic signal.

We regarded a significant positive correlation between male and female variables as corroborating the prediction of sexual coevolution. We assessed the intersexual correlation using all variables simultaneously by phylogenetic canonical correlation analysis (pCCA) [26,36] with variables categorized by sex. We also conducted bivariate phylogenetic regressions analyses (pRA) [26] between all pairs of male and female variables.

The hypothesis that solicitous courtship and precopulatory antagonism operate in a mutually exclusive manner predicts that species should be distributed in two distinct clusters. The prediction was assessed using several methods. Cluster analysis was performed using a model-based approach [37] implemented in *mclust* [38], which evaluates results from different clustering models using the Bayesian Information Criterion (BIC). Recovery of two unmixed clusters would corroborate the distinctiveness of solicitous courtship and precopulatory antagonism. We also assessed whether interspecific variation was distributed continuously or clustered into functional groups by examining species scores from the pCCA, pRAs and pPCA [26], although these methods are not optimized for group classification [39].

We tested the prediction that species with penile sacs and unarmed female genital opercula occupy a distinct solicitous group, and that those without penile sacs but with female pregenital armature occupy a distinct antagonistic category. We used phylogenetic flexible discriminant analysis [40] and standard (non-phylogenetic) linear discriminant analysis in the *MASS* package [41] for one set of analyses using presence or absence of penile gift-bearing sacs as the grouping variable and another set of analyses using presence or absence of female pregenital barriers.

## Results

### (a) Effectiveness of dimensional size correction

**Fig 3:**
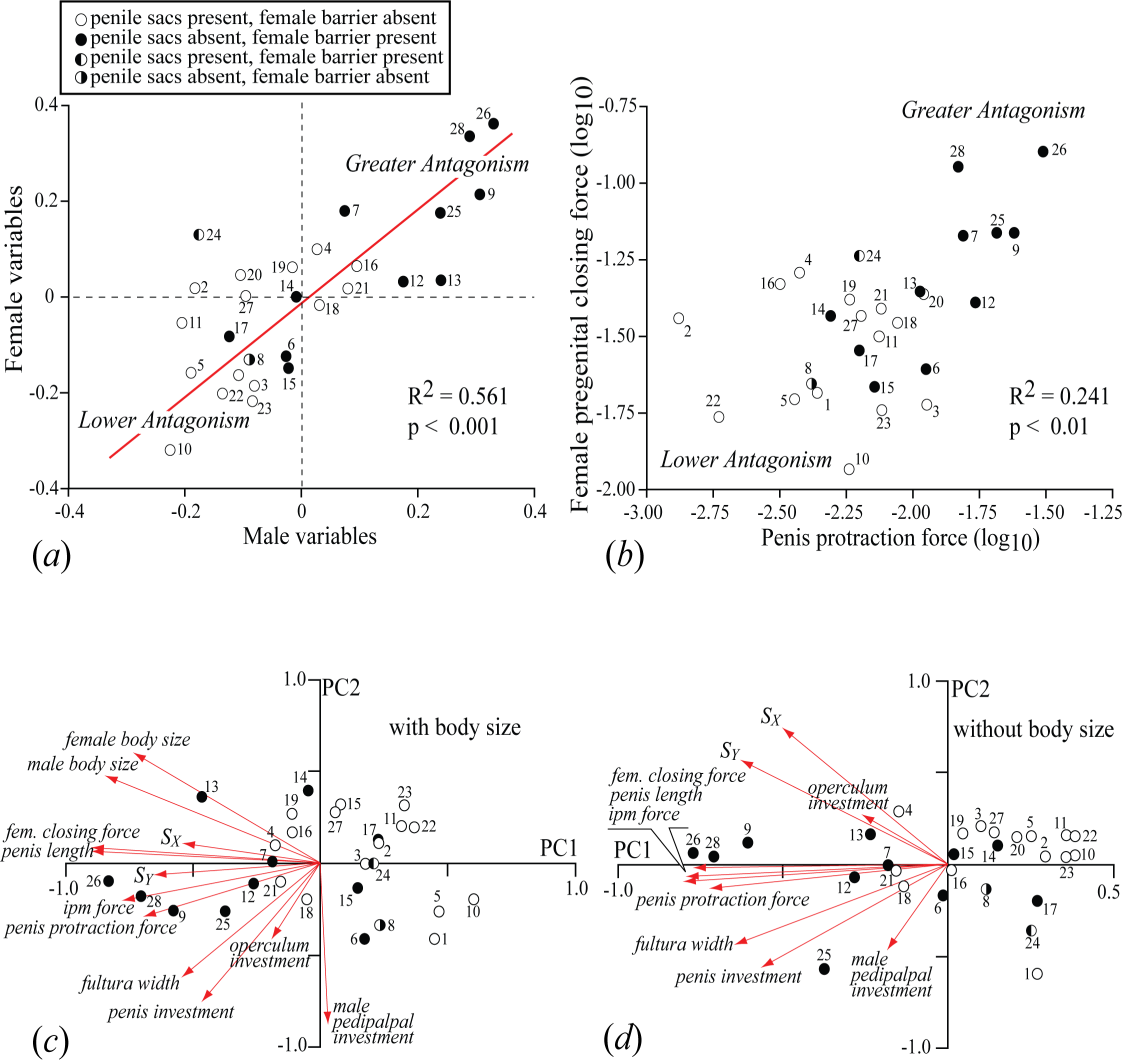
Evidence for sexual coevolution among reproductive traits and arrangement of species along a spectrum of mechanical antagonistic potential. (*a*) Species scores on canonical function 1 from phylogenetic canonical correlation analysis of eight male and two female mechanical variables. (*b*) Bivariate phylogenetic regression of relative closing force of the female pregenital opening, and relative protraction force of the penis. (*c*) Phylogenetic principal components analyses (pPCA) of all variables, including male and female body size(PCs 1 and 2: 57.52% variance, λ = 6.75e-5, lnL λ = 175), demonstrating that PC1 is not the size axis. (*d*) pPCA of all reproductive variables without body size. See Table 2 for PC loadings and other details of PCAs.

We compared results from a phylogenetic principal components analysis (pPCA) with body size and a pPCA without body size to assess the effectiveness of dimensional size correction (Fig 3*C* and 3*D*). Male and female body size did not load exclusively on PC1 (Table 2), indicating that PC1 is not simply a size axis. Phylogenetic regression analyses (pRA) of male and female variables using dimensional size correction produced results very similar to those obtained from pRA using regression-based size correction. The pRA using the dimensional approach yielded significant correlations between female pregenital closing force and each of three male variables—penis protraction force (R^2^ = 0.241, p<0.01) (Fig 3*B*), intrinsic penile muscle force (R^2^ = 0.264, p<0.01) and penis length (R^2^ = 0.482, p<0.0001) (S4 Fig)—but pRAs for other variable pairs, such as cuticular investment in male and female structures, were not significant. The pRA using phylogenetic residual size correction found significant correlations between the same pairs of female and male variables—female pregenital closing force vs. penis protraction force (R^2^ = 0.311, p<0.01), intrinsic penile muscle force (R^2^ = 0.251, p<0.01) and penis length (R^2^ = 0.142, p<0.05)—but no other variable pairs. We concluded that the dimensional approach to size correction was effective.

**Table 2:** Trait loadings of phylogenetic principal component analyses with and without body size, eigenvalues, and percent variance explained by first two principal components.

	Phylogenetic Principal Components Analysis			
	with size		without size	
Trait	PC1	PC2	PC1	PC2
Male body size	-0.823	0.467		
Female body size	-0.685	0.598		
Female pregenital closing force	-0.883	0.044	-0.796	-0.039
Intrinsic penis muscle force	-0.719	-0.181	-0.771	-0.006
Penis protractor muscle force	-0.658	-0.252	-0.727	-0.115
Penis length	-0.883	0.066	-0.806	-0.070
Penis investment	-0.434	-0.660	-0.570	-0.547
Male pedipalpal investment	0.016	-0.791	-0.185	-0.457
Female operculum investment	-0.154	-0.307	-0.268	0.298
Penis section modulus (*S*_*X*_)	-0.502	0.103	-0.501	0.776
Penis section modulus (*S*_*Y*_)	-0.600	-0.067	-0.633	0.599
Fultura width	-0.514	-0.555	-0.652	-0.424
**Eigenvalue**	4.746	2.157	3.914	1.756
**% Variance**	39.55	17.97	39.14	17.56

### (b) Tests of sexual coevolution in mechanical variables

A phylogenetic canonical correlation analysis (pCCA) using all 10 variables partitioned by sex (λ = 6.75e-05, lnL λ = 43.55) yielded two canonical factors, with only CF1 offering useful information (Fig 3*A*). Major-axis regression of the CF1 axes revealed a significant correlation (R^2^ = 0.561, p< 0.001) consistent with sexual coevolution. Species were distributed continuously along the regression, with "high antagonistic" species occupying one end, "low antagonistic" species occupying the other, and a central region of overlap. Those species with female pregenital armature and lacking penile gift sacs occurred in the "high antagonistic" region; those with penile gift sacs and lacking female armature were concentrated in the "low antagonistic" region. Phylogenetic regression analyses (pRA) using dimensional size correction yielded significant correlations between female pregenital closing force and each of three male variables—penis protraction force (Fig 3*B*), intrinsic penile muscle force (S4*A* Fig), and penis length (S4*B* Fig)—which is consistent with sexual coevolution between these variables. These regressions showed species distributions similar to that recovered by pCCA.

Results from pPCA provided several significant insights. We interpreted PC1 to be an axis of mechanical antagonistic potential. Species were arranged along this axis with "high antagonism" forms concentrated toward one end, "low antagonism" forms at the other, and a region of overlap in the center. Further, the distribution of species is very similar to that recovered by pCCA. Those variables that were most highly correlated with one another in pairwise pRA—all estimated male and female force variables and penis length—loaded almost exclusively on PC1 (Table 2), with the force variables being perhaps the most direct indictor of precopulatory antagonistic ability. Cuticular variables—fultura width, penile flexural strength, and cuticular investment variables—loaded weakly to moderately on both PC1 and PC2 (Table 2).

### (c) Clustering, discriminant function analysis and categories of precopulatory strategies

Model-based clustering analysis using BIC from mixture models was fitted by an EM algorithm [38]. The best model for species clustering based on BIC was an ellipsoidal multivariate normal model with one cluster (lnL = 174.93, BIC = 49.96), a result that was inconsistent with the prediction that species are distributed into separate solicitous and antagonistic categories. Separate solicitous and antagonistic clusters were also absent from plots derived from pCCA, pPCA and pRA (Fig 3).

Standard linear discriminant analysis (LDA) resulted in 85% of species being placed into anticipated solicitous and antagonistic groups based on presence and absence of penile sacs, respectively (S5 Fig). Phylogenetic flexible discriminate analysis (pFDA), which incorporated an estimate of Pagel's lambda (λ = 6.75e-05) obtained from pPCA, grouped species at an error rate of 12.4% based on penile morphology. Using presence or absence of female pregenital armature, LDA correctly placed 89% of species, which was consistent with results derived from pFDA (mean error rate: 10%). However, neither the penile-sacs grouping variable (Wilk’s λ = 0.44, F_12,15_ = 1.56, p = 0.2054) nor the female pregenital barrier variable (Wilk’s λ = 0.46, F_12,15_ = 1.47, p = 0.2387) provided a statistically significant discriminant model. The inability of cluster and discriminant analyses to partition species into separate groups is inconsistent with the prediction that leiobunine harvestmen present two mutually-exclusive precopulatory strategies. Rather, results from discriminant analysis, pCCA, pRA and pPCA indicate the species are distributed continuously.

## Discussion

### (a) Sexual coevolution of functional genitalic traits and a spectrum of precopulatory antagonism

Our previous research on the leiobunine harvestmen of eastern North America [15] suggests that genitalic diversity is consistent with two sexually coevolved and mutually-exclusive precopulatory strategies: 1) a primitive solicitous strategy in which males bear nuptial gifts in cuticular penile sacs and females have unarmed pregenital openings, and 2) a derived antagonistic strategy in which males lack penile sacs and females have sclerotized pregenital defenses. Two aspects of that study led us to question whether its conclusions would be robust to new morphological data. First, the analysis was based on the phylogenetic distribution of two binary (categorical) characters that biased the result toward recovery of up to four mutually-exclusive character-state combinations. Second, any causal link between the loss of penile sacs and gain of pregenital barriers is likely to be indirect; that is, the loss of penile sacs does not seem necessary for males to apply mechanical forces to the female's pregenital opening, and females need not defend their pregenital openings against the absence of male-generated nuptial gifts. The present analysis addressed these issues by testing predictions using continuously-distributed functional variables, most derived from biomechanical interpretations of genitalic structure.

The prediction that continuous functional variables have undergone sexual coevolution was largely corroborated by our analysis. Phylogenetic canonical correlation (pCCA), with variables partitioned by sex, yielded a significant positive correlation between the two female and eight male variables (Fig 3*A*). Furthermore, bivariate phylogenetic regression analysis (pRA) between male and female variables produced significant positive correlations in comparisons of relative female pregenital closing force and three male variables—relative penis protraction force, relative intrinsic penile muscle force, and penis length (Fig 3*B* and S4 Fig). These variables have direct mechanical interactions (i.e., synergism among male variables and antagonism between male and female variables) and also load heavily and almost exclusively on PC1 (interpreted here as an axis of antagonistic potential) in phylogenetic principal components analysis (pPCA) (Table 2 and Fig 3*D*). Relationships between all other pairs of male and female variables were not significant, although they may have contributed to the pCCA results. The non-significant bivariate relationships involved cuticular variables—penile fulturae, penile section moduli, and cuticular investment in the penis, female operculum, and male pedipalp. It is possible that their anticipated role in antagonism was too indirect or oversimplified; direct measurement of their kinetic properties might have been more informative than the structural proxies used here. We conclude that sexual coevolution exists between functional variables collectively and between pairs of variables associated most directly with the production of precopulatory antagonistic forces (i.e., attempted penetration of the female pregenital opening by males and resistance by females).

Cluster analysis of functional variables did not organize species into predicted solicitous and antagonistic groups, or into separate, quantitatively defined groups of any kind. In addition, discriminant function analyses—some using presence or absence of penile sacs and some using presence or absence of female pregenital barriers as grouping variables—failed to place species reliably into two classes (S5 Fig). In contrast, pCCA, pPCA and the significant pRAs arranged species into similar and essentially linear distributions (Fig 3*A*, 3*B* and 3*D*). We attribute this distribution to a spectrum of precopulatory antagonistic potential, a conclusion supported by two main observations. First, there is a consistent and intuitively sensible polarity in the distribution of species. Specifically, "antagonistic" species (i.e., females have forceful pregenital closing mechanisms and pregenital barriers and males have long, non-sacculate penes operated by forceful intrinsic and protractor muscles) tend to occur at one end of the distribution, "solicitous" species (i.e., females have weak pregenital closing mechanisms and no pregenital barriers and males have short, sacculate penises operated by low-force mechanisms) are located at the other end of the distribution, and there is a central region of overlap, which includes species with unusual intermediate combinations of features (e.g., female pregenital barrier and short sacculate penes). Second, the species are distributed broadly along PC1 in the pPCA and the variables that load most heavily on this axis are those most closely associated with antagonistic interactions between the sexes, especially estimated relative mechanical forces. These observations lead us to conclude that PC1 represents an axis of precopulatory antagonistic potential. The continuous distribution of species along the antagonistic axis suggests that extant genitalic diversity reflects variable combinations of intensity of solicitation and antagonism, that mating systems can evolve gradually along the solicitation-to-antagonism species, and that antagonism can be quantified by individual or latent variables, especially estimated force variables.

### (b) Can we understand genitalic diversification in harvestmen using comparative methods?

Behavioral ecologists have tended to attribute the origin and maintenance of certain reproductive traits to specific evolutionary processes. For example, the presence of apparent nuptial gifts or male ornaments has generally been associated with female choice [3], while apparent sexual armaments or antagonistic intersexual behaviors have implied the work of sexual conflict [42]. If such associations were valid, results from the present analysis might indicate that female choice and sexual conflict have acted together at different intensities in harvestmen to generate a spectrum of genitalic morphologies. However, empirical and theoretical work on female mate choice shows that these associations are unreliable (e.g., nuptial gifts may manipulate female physiology in ways that lower her fitness) [43], that female choice and sexual conflict offer non-exclusive morphological and behavioral predictions (see below), and even that choice and conflict themselves are inseparable rather than alternative processes [7,9]. The complicated and unsettled status of the discipline introduces substantial difficulties for partitioning effects of different evolutionary mechanisms at the population level [4,8,44], let alone at phylogenetic timescales.

Due to the correlative nature of comparative methods, progress using phylogeny-based tests relies on evolutionary hypotheses that predict mutually-exclusive patterns of interspecific diversity. Current concepts of female choice and sexual conflict fail to offer such predictions and, in fact, blur the distinction between sexual ornaments and armaments. For example, the Fisherian "run-away" model of female choice [44,45] maintains that mating enhances fitness in both sexes and predicts that attractive male features and female preference for these features will be represented disproportionately in the next generation, leading to an evolutionary progression of increasingly complex or exaggerated male ornaments. However, the same progression is also consistent with conflict-based "chase-away" models [46,47], wherein male attractiveness entices females to mate at higher than optimal rates, thereby increasing male fitness at the expense of female fitness. Females would then be selected to evolve higher sensitivity thresholds, thereby escaping male exploitation but also increasing selective pressure on males to intensify enticing stimuli [46]. An evolutionary progression in male attractiveness is thus consistent with both female choice and sexual conflict. Similarly, armaments and antagonistic behaviors may evolve via sexual conflict; for example, males may enhance individual fitness by coercing copulations in a manner that reduces female fitness, either by overriding mating preferences or reducing future reproductive potential [8,48]. However, female resistance to copulation and male efforts to overcome this resistance may be an inevitable consequence of female choice; that is, low-quality males that cannot overcome female resistance will be excluded in favor of high-quality males that can. Under this scenario, fitness benefits could accrue to females directly by reducing a potentially detrimental mating rate or indirectly via production of antagonistically-superior sons [8,9]. Thus, apparent sexually antagonistic coevolution is consistent with sexual conflict, female choice, or both [8,9,49]. In sum, comparative methods may discover evolutionary patterns in genitalic form and function but are unlikely to distinguish potential roles for female choice and sexual conflict in producing them.

If the predictions of individual-, generational- or population-level models of genitalic diversification, like female choice and sexual conflict, cannot be usefully extrapolated to phylogenetic scales, one must ask whether there are conditions under which phylogenetic comparative methods can contribute to understanding genitalic evolution. We maintain that there are. For example, the classic work of Emlen & Oring [50] highlighted the role of ecology and life history in limiting sexual selection and the structure of mating systems (e.g., polygamy requires access to spatial, temporal and nutritional resources sufficient to find and acquire multiple mates). If genital morphology is likewise influenced by sexual selection, as is widely accepted [2,4,6] and even demonstrated in the case of genital complexity and polygamy [11], one might expect interspecific associations between ecology and genitalic structure [51]. Such associations may be too indirect for comparative methods to detect in many systems, but harvestman genitalia appear to be a particularly apt case. Penes in leiobunine harvestmen are involved directly in the delivery of secreted nuptial gifts produced from material acquired in the environment, and antagonistic skeletomuscular mechanisms impose potential costs in material, energy, and developmental time. Interestingly, leiobunine harvestmen in tropical areas have "low-antagonism" morphologies (penile gift sacs, no female pregenital barriers, short penes, etc.), whereas "high-antagonism" morphologies are limited to temperate regions. These associations suggest that the intensity of precopulatory antagonism are inversely correlated with the duration or quality of the breeding season; that is, antagonism may be favored over enticement when resources are in short supply or of unpredictable duration [5,16]. Significantly, such predictions can be tested at phylogenetic scales, where species may differ substantially in ecology or life history, and within species, where relationships between ecology, sexual selection, and genital function can be explored via observation, experiment, or comparisons between populations occupying different environments. Consequently, hypotheses formulated for use with comparative methods may offer explanations for genitalic diversification that are more robust to evolutionary timescale than those designed for population-level tests.

## Supporting Information

S1 Fig Female genital anatomy of leiobunine harvestmen. (*a*) Ventral view of intact female *Leiobunum verrucosum* showing proximity of mouth and pregenital opening. (*b*) Ventral view of female *L*. *verrucosum* with genital operculum removed and reflected to show lack of opercular and sternal armature at pregenital opening. (*c*) Ventral view of female *Hadrobunus maculosus* with genital operculum removed and reflected to show opercular and sternal armature of the pregenital opening. (*d*) Interaction of opercular and sternal armature to form pregenital barrier in female *H*. *maculosus*. (*e*) Ventral view of female *L*. *hoffmani* with genital operculum removed and reflected to show opercular and sternal armature. (f) Interaction of opercular and sternal armature to form pregenital barrier in female *L*. *hoffmani*.
(TIF)

S2 Fig Method for inferring maximum relative female pregenital closing force. (*a*) Mid-sagittal section of female *L*. *verrucosum* showing position of operculum levator (pregenital closing) muscle. (*b*) Relative closing force of levator muscle was estimated by determining muscle scar width (*w*) in millimeters and fiber-attachment angle (*θ*) in degrees at six evenly spaced points (*w*_1_-*w*_6_) along the levator muscle scar of the operculum. (*c*) Using GraphPad Prism, v. 5.04 (GraphPad Software, San Diego, Calif., USA), the values (*w*_n_ x cos *θ*_n_) were plotted against muscle-scar length and fitted using a least-squares polynomial regression. The resulting equation was integrated over the interval 0 to total scar length to estimate the maximum relative closing force produced by the muscle (*F*_*I*_). Because the genital operculum is a lever system, the relative closing force at the anterior margin (*F*_*O*_) equals *F*_*I*_ multiplied by the muscle’s mechanical advantage (*L*_*I*_/*L*_*O*_). *L*_*I*_ is the distance from the hinge to the point where *F*_*I*_ is applied, which is taken as the longitudinal position of the centroid of *F*_*I*_ (i.e., the point along the muscle scar where the cumulative area under the regression curve equals *F*_*I*_/2). *L*_*O*_ is the distance from the hinge (fulcrum) to the anterior margin of the operculum and was measured directly. (d) Method for estimating relative force of the intrinsic penile muscle. The effective relative force of the muscle (*F*_*I*_) is calculated as (*n* x cos *θ* x *a*), where *n* is fiber number, *θ* is the average fiber angle with respect to the tendon and *a* is the average fiber cross-sectional area, The area *a* is calculated as π(0.5*d*)^2^, where *d* is average fiber diameter. The relative force exerted by the muscle at the tip of the penis is *F*_*I*_ multipled by the mechanical advantage of the muscle at the glans-shaft joint, Mechanical advantage is *L*_*O*_/*L*_*I*_, where *L*_*O*_ is the length of the glans and *L*_*I*_ is the height of the joint.
(TIF)

S3 Fig Penes and cross-sections from a sample of leiobunine species. Examples are displayed for males of four species: *Leiobunum ventricosum* (sacculate), *L*. *crassipalpe* (non-sacculate), *Hadrobunus* n. sp. 1 (non-sacculate), and *L*. *bracchiolum* (sacculate). High-contrast images of penile cross-sections (on left) were generated in order to estimate section modulus (*S*_*X*_, *S*_*Y*_), which are associated with flexural strength. X and Y axes of cross-section are indicated. Dorsal perspectives of penes are shown on the right. Scale applies to whole penes only.
(TIF)

S4 Fig Bivariate phylogenetic regressions of dimensionally size-corrected data. (*a*) Maximum relative closing force of the female pregenital opening versus penis length. (*b*) Maximum relative closing force of female pregenital opening versus maximum relative intrinsic penile muscle force.
(TIF)

S5 Fig Histograms of discriminant classification. Species scores on linear discriminant function 1 based on all biomechanical data under two sets of grouping variables: (*a*) penile sac presence or absence and (*b*) female pregenital barrier presence or absence.
(TIF)

S1 Table Sample data. (DOCX)
