# Supplementary figures and images for: Biomechanical Diversity of Mating Structures among Harvestmen Species Is Consistent with a Spectrum of Precopulatory Strategies
Source: PLoS One. 2015 Sep 9;10(9):e0137181. doi: 10.1371/journal.pone.0137181 (PMC4564142; doi:10.1371/journal.pone.0137181)

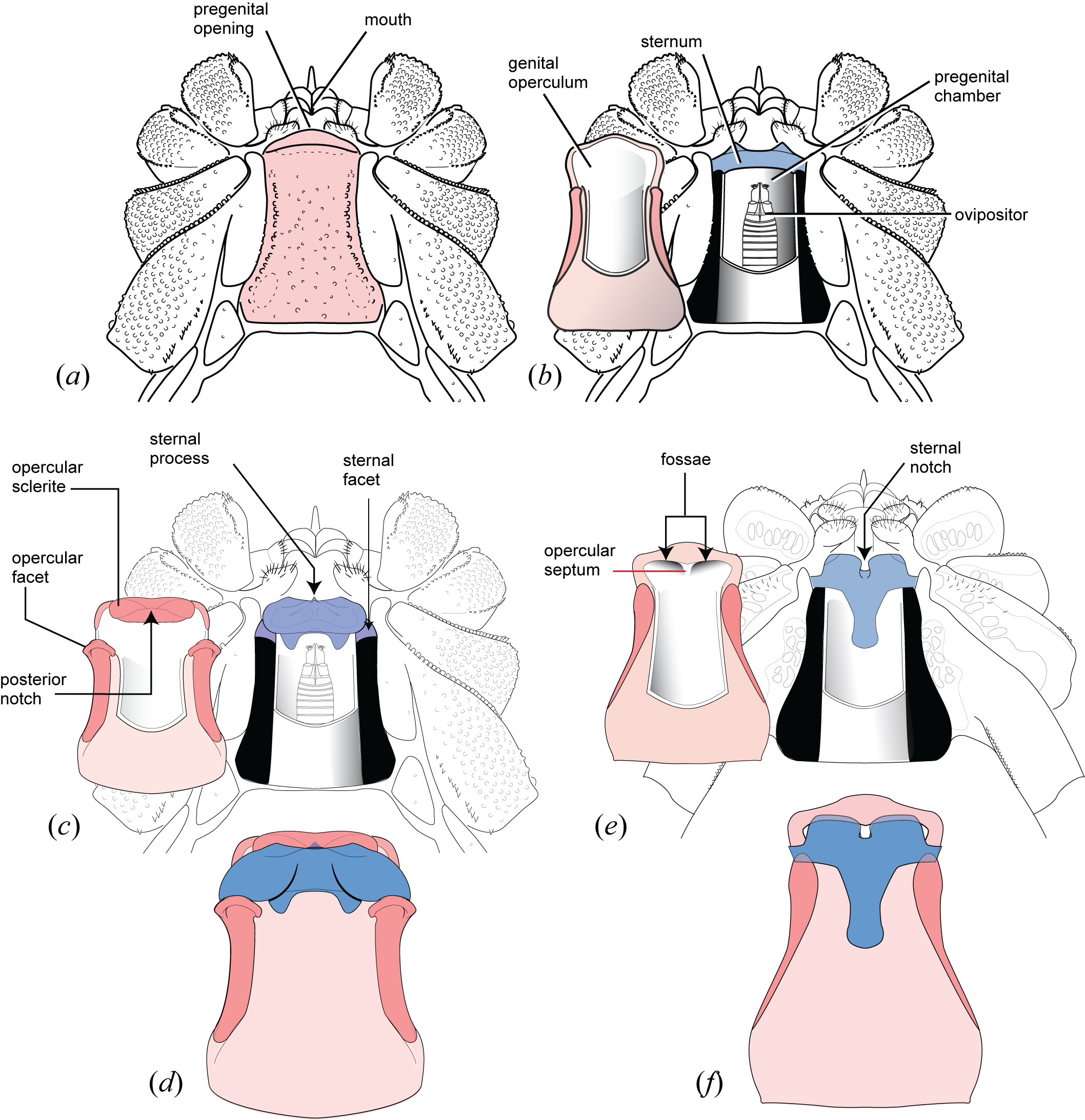

Supplement: S1 Fig — (a) Ventral view of intact female Leiobunum verrucosum showing proximity of mouth and pregenital opening. (b) Ventral view of female L. verrucosum with genital operculum removed and reflected to show lack of opercular and sternal armature at pregenital opening. (c) Ventral view of female Hadrobunus maculosus with genital operculum removed and reflected to show opercular and sternal armature of the pregenital opening. (d) Interaction of opercular and sternal armature to form pregenital barrier in female H. maculosus. (e) Ventral view of female L. hoffmani with genital operculum removed and reflected to show opercular and sternal armature. (f) Interaction of opercular and sternal armature to form pregenital barrier in female L. hoffmani. (TIF) [file pone.0137181.s001.tif]

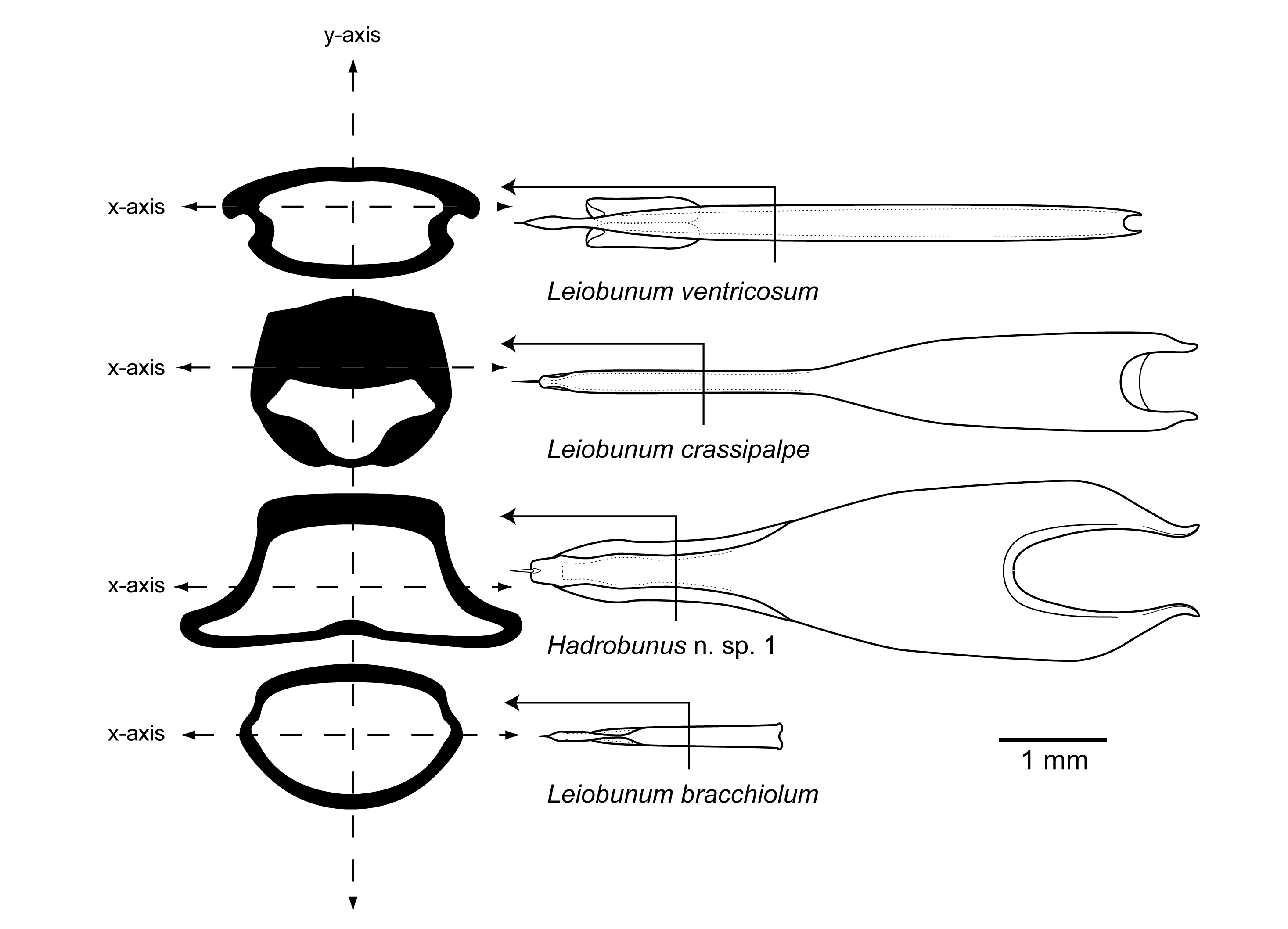

Supplement: S2 Fig — (a) Mid-sagittal section of female L. verrucosum showing position of operculum levator (pregenital closing) muscle. (b) Relative closing force of levator muscle was estimated by determining muscle scar width (w) in millimeters and fiber-attachment angle (θ) in degrees at six evenly spaced points (w 1-w 6) along the levator muscle scar of the operculum. (c) Using GraphPad Prism, v. 5.04 (GraphPad Software, San Diego, Calif., USA), the values (w n x cos θ n) were plotted against muscle-scar length and fitted using a least-squares polynomial regression. The resulting equation was integrated over the interval 0 to total scar length to estimate the maximum relative closing force produced by the muscle (F I). Because the genital operculum is a lever system, the relative closing force at the anterior margin (F O) equals F I multiplied by the muscle’s mechanical advantage (L I/L O). L I is the distance from the hinge to the point where F I is applied, which is taken as the longitudinal position of the centroid of F I (i.e., the point along the muscle scar where the cumulative area under the regression curve equals F I/2). L O is the distance from the hinge (fulcrum) to the anterior margin of the operculum and was measured directly. (d) Method for estimating relative force of the intrinsic penile muscle. The effective relative force of the muscle (F I) is calculated as (n x cos θ x a), where n is fiber number, θ is the average fiber angle with respect to the tendon and a is the average fiber cross-sectional area, The area a is calculated as π(0.5d)2, where d is average fiber diameter. The relative force exerted by the muscle at the tip of the penis is F I multipled by the mechanical advantage of the muscle at the glans-shaft joint, Mechanical advantage is L O/L I, where L O is the length of the glans and L I is the height of the joint. (TIF) [file pone.0137181.s002.tif]

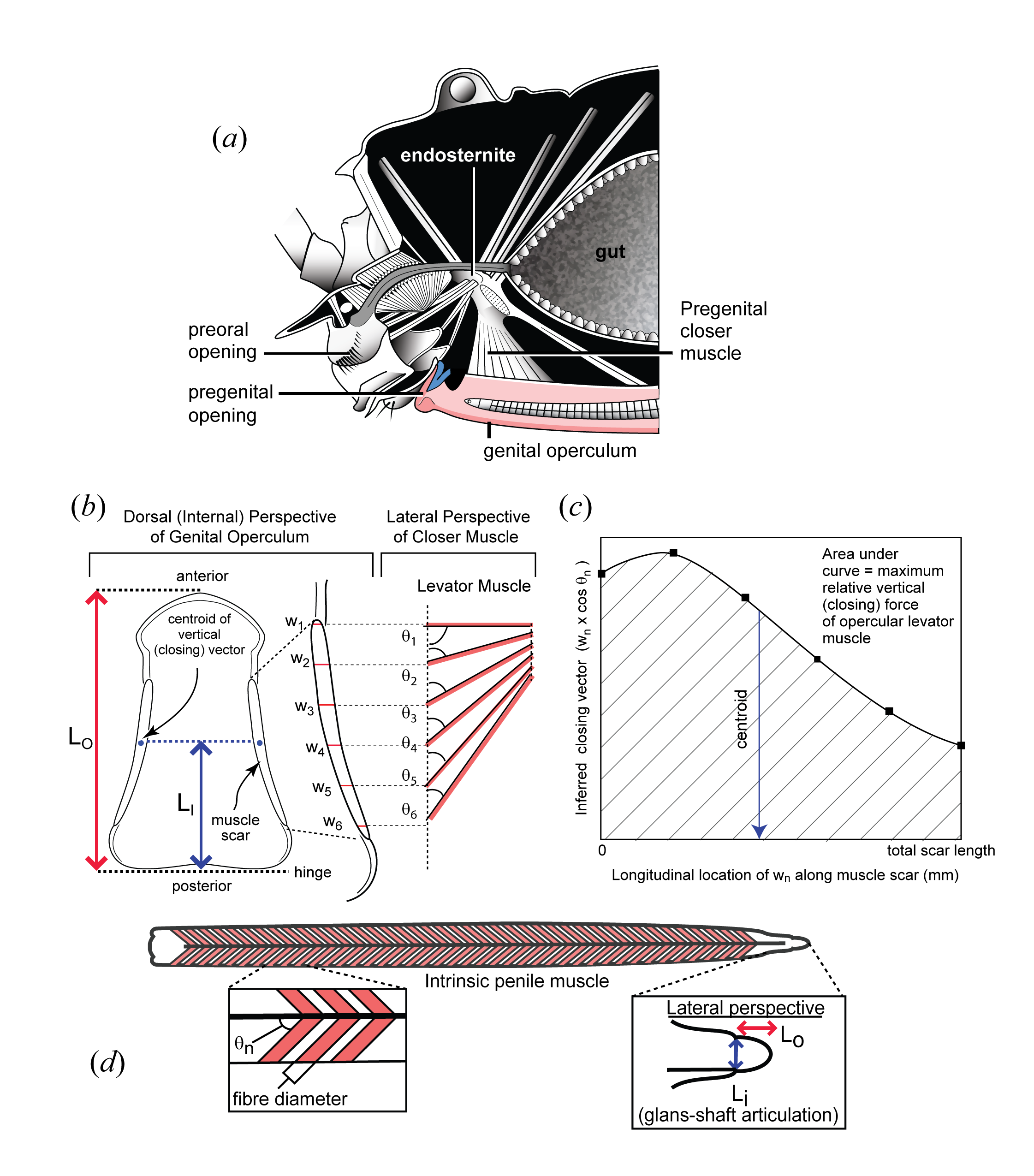

Supplement: S3 Fig — Examples are displayed for males of four species: Leiobunum ventricosum (sacculate), L. crassipalpe (non-sacculate), Hadrobunus n. sp. 1 (non-sacculate), and L. bracchiolum (sacculate). High-contrast images of penile cross-sections (on left) were generated in order to estimate section modulus (S X, S Y), which are associated with flexural strength. X and Y axes of cross-section are indicated. Dorsal perspectives of penes are shown on the right. Scale applies to whole penes only. (TIF) [file pone.0137181.s003.tif]

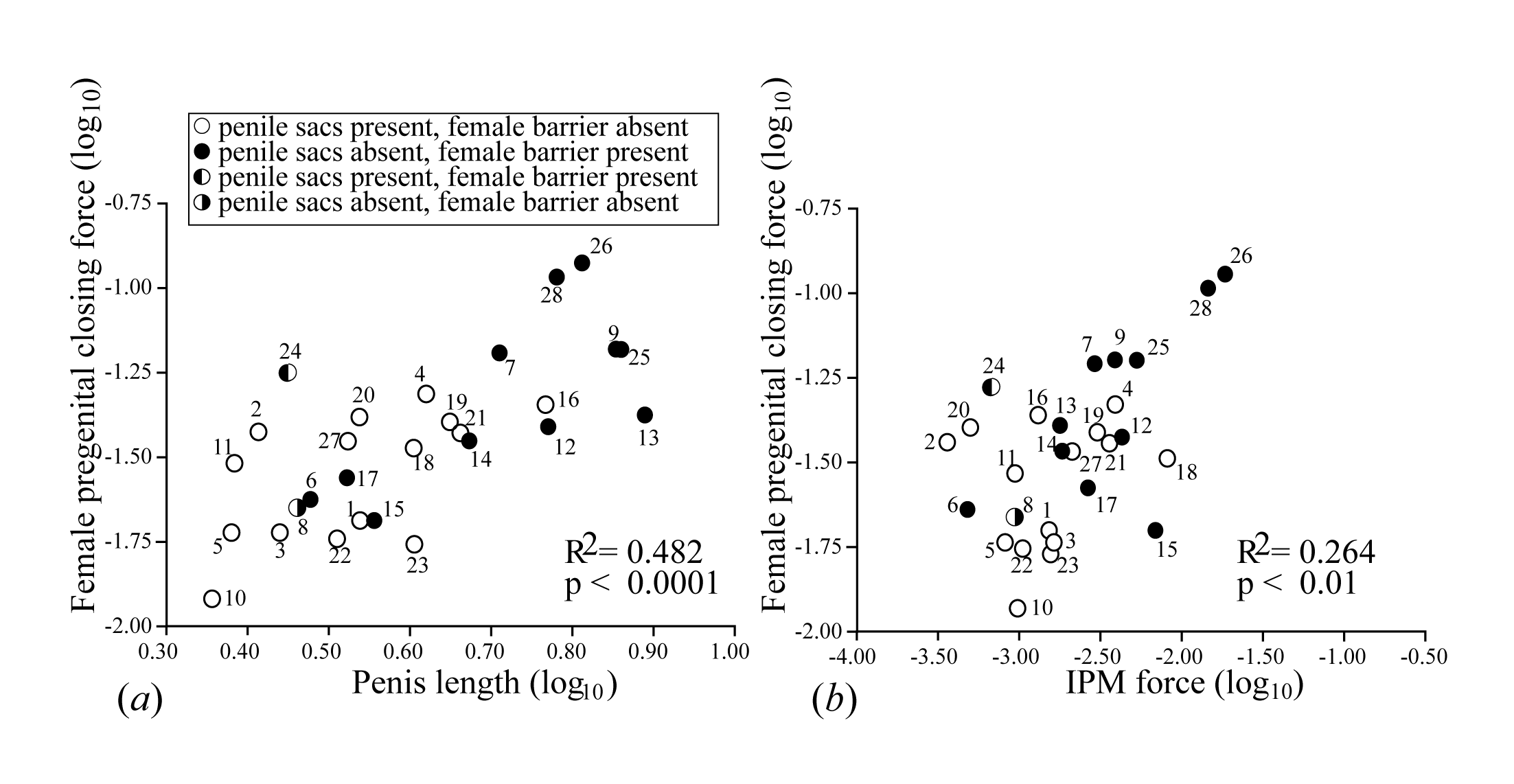

Supplement: S4 Fig — (a) Maximum relative closing force of the female pregenital opening versus penis length. (b) Maximum relative closing force of female pregenital opening versus maximum relative intrinsic penile muscle force. (TIF) [file pone.0137181.s004.tif]

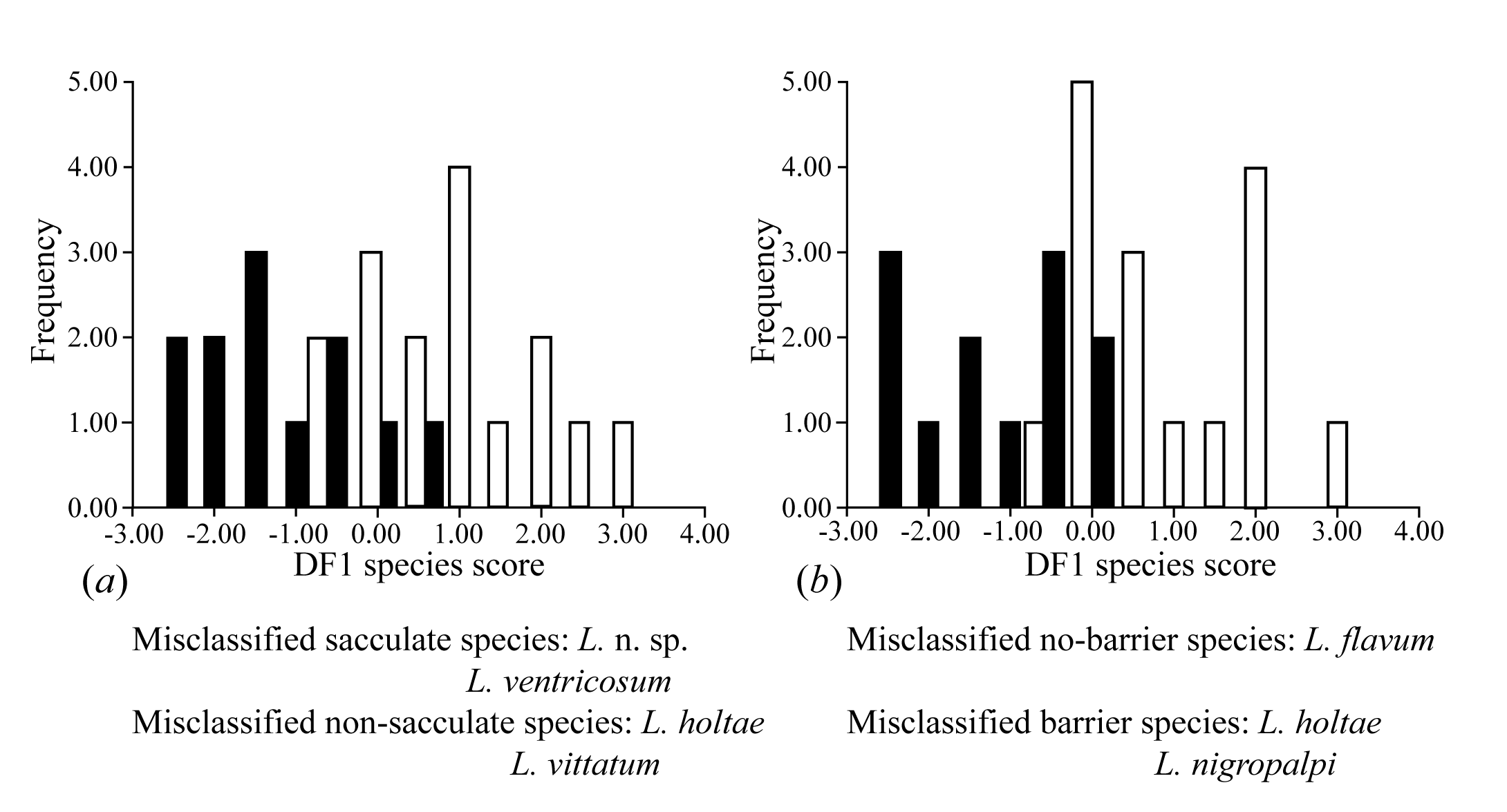

Supplement: S5 Fig — Species scores on linear discriminant function 1 based on all biomechanical data under two sets of grouping variables: (a) penile sac presence or absence and (b) female pregenital barrier presence or absence. (TIF) [file pone.0137181.s005.tif]
