# Supplementary material for: Biomechanical Diversity of Mating Structures among Harvestmen Species Is Consistent with a Spectrum of Precopulatory Strategies
Source: PLoS One. 2015 Sep 9;10(9):e0137181. doi: 10.1371/journal.pone.0137181 (PMC4564142; doi:10.1371/journal.pone.0137181)
Supplement: S1 Table — (DOCX) [file pone.0137181.s006.docx]

**Electronic Supplementary Materials**

**Biomechanical diversity of mating structures among harvestmen species is consistent with a spectrum of precopulatory strategies**

 Mercedes M. Burns^1#a*^ and Jeffrey W. Shultz^1^

^1^ *BEES Program and Department of Entomology, University of Maryland, College Park, MD, USA*

^#a^ *Department of Biology, San Diego State University, San Diego, CA, USA*

**S1 Table. Sample data.**

| **Species** | **GenBank**  **accession numbers** | **Molecular specimen locality** | **Morphological specimen locality** | **Number of specimens examined** | | |
| --- | --- | --- | --- | --- | --- | --- |
|  |  |  |  | **Male morphology (except *S_X_*,S_Y_)** | **Female morphology** | **Penis section modulus (*S_X_*,S_Y_)** |
| *Eumesosoma roeweri* | JQ432365,  JQ432307, JQ432253 | USA: TX: Wichita Co. | USA: TX: Travis Co., USA: TX: Wichita Co.,  USA: TX: Williamson Co. | 6 | 5 | 3 |
| *Hadrobunus grandis* | JQ432358, JQ432300, JQ432249 | USA: FL: Alachua Co. | USA: FL: Alachua Co. | 4 | 5 | 1 |
| *Hadrobunus* n.sp.1 | JQ432359, JQ432301, JQ432250 | USA: TN: Sevier Co. | USA: TN: Blount Co.,  USA: WV: Monongalia Co. | 6 | 5 | 4 |
| *Hadrobunus* *maculosus* | JQ432360, JQ432302,  JQ432361, JQ432362, JQ432303, JQ432304, JQ432251 | USA: MD: Howard Co. | USA: MD: Garrett Co. | 6 | 5 | 5 |
| *Hadrobunus* n.sp. 2 | JQ432364, JQ432306, JQ432252 | USA: MO: Ozark Co. | USA: KS: Douglas Co. | 2 | 6 | 3 |
| *Leiobunum aldrichi* | GQ870650, JQ432342, JQ432284, GQ872154,  GQ870649, JQ432343, JQ432285, GQ872153,  JQ432344, JQ432286, JQ432238 | USA: MI: Calhoun Co. | USA: OH: Stark Co. | 5 | 5 | 2 |
| *Leiobunum bimaculatum* | JQ432366, JQ432308 | USA: FL: Jackson Co. | USA: VA: Nansemond Co.,  USA: MS: George Co.,  USA: GA: Toombs Co.,  USA: GA: Tyton Co. | 2 | 6 | 2 |
| *Leiobunum*  *bracchiolum* | JQ432330, JQ432272, JQ432230 | USA: NC: Guilford Co. | USA: MD: Frederick Co.,  USA: MD: Prince Georges Co. | 2 | 6 | 2 |
| *Leiobunum calcar* | GQ870653, JQ432316, JQ432258, GQ872157,  JQ432317, JQ432259, JQ432223,  JQ432319, JQ432261,  GQ870655, JQ432320, JQ432262, GQ872158,  JQ432318, JQ432260 | USA: MD: Frederick Co. | USA: NC: Madison Co.,  USA: MD: Garrett Co. | 4 | 8 | 4 |
| *Leiobunum crassipalpe* | JQ432331, JQ432273,  JQ432332, JQ432274, JQ432231 | USA: MO: Butler Co. | USA: MO: Butler Co. | 2 | 5 | 2 |
| *Leiobunum euserratipalpe* | JQ432321, JQ432263,  GQ870656, JQ432322, JQ432264, GQ872160 | USA: MD: Montgomery Co. | USA: PA: Bucks Co. | 5 | 7 | 3 |
| *Leiobunum flavum* | JQ432353, JQ432295, JQ432245 | USA: AR: Garland Co. | USA: AR: Garland Co. | 5 | 3 | 3 |
| *Leiobunum formosum* | JQ432354, JQ432296,  JQ432356, JQ432298, JQ432247,  JQ432355, JQ432297, JQ432246,  JQ432357, JQ432399, JQ432248 | USA: FL: Jackson Co. | USA: FL: Hernando Co.,  USA: VA: Dickerson Co.,  USA: VA: Northampton Co. ,  USA: FL: Liberty Co. | 5 | 4 | 6 |
| *Leiobunum hoffmani* | GQ870654, JQ432315, JQ432257, GQ872159 | USA: VA: Grayson Co. | USA: VA: Grayson Co. | 6 | 6 | 3 |
| *Leiobunum holtae* | JQ432345, JQ432287, JQ432239,  JQ432346, JQ432288, JQ432240 | USA: TN: Cumberland Co. | USA: TN: Hamilton Co.,  USA: TN: Van Buren Co. | 2 | 3 | 2 |
| *Leiobunum* n.sp. 1 | JQ432352, JQ432294 | USA: NE: Lancaster Co. | USA: NE: Lancaster Co. | 5 | 5 | 4 |
| *Leiobunum nigropalpi* | JQ432323, JQ432265, JQ432224,  JQ432324, JQ432266, JQ432225,  JQ432325, JQ432267, JQ432226 | USA: MD: Frederick Co. | USA: MD: Garrett Co. | 6 | 11 | 4 |
| *Leiobunum politum* | JQ432326, JQ432268, JQ432227,  JQ432327, JQ432269, JQ432228,  JQ432328, JQ432270, JQ432229,  JQ432329, JQ432271 | USA: AR: Lawrence Co. | USA: MO: Greene Co.,  USA: MO: Butler Co.,  USA: AR: Lafayette Co. | 2 | 4 | 1 |
| *Leiobunum potosum* | JQ432370, JQ432312 | MEXICO: Tlaxcala, Ixtacuixtla | MEXICO: Puebla,  MEXICO: Guerrero | 6 | 5 | 2 |
| *Leiobunum relictum* | JQ432340, JQ432341, JQ432282, JQ432283, JQ432237 | USA: OK: Comanche Co. | USA: OK: Comanche Co. | 3 | 6 | 2 |
| *Leiobunum royali* | JQ432367, JQ432309, JQ432254 | MEXICO: Veracruz, Xalapa | MEXICO: Veracruz | 5 | 6 | 2 |
| *Leiobunum townsendi* | JQ432369, JQ432311 | USA: AZ: Cochise Co. | USA: AZ: Cochise Co. | 5 | 5 | 2 |
| *Leiobunum uxorium* | JQ432339, JQ432281, JQ432235,  JQ432338, JQ432280, JQ432236 | USA: VA: Smythe Co. | USA: VA: King George Co.,  USA: PA: Lancaster Co.,  USA: MD: Howard Co.,  USA: PA: Cumberland Co. | 3 | 8 | 2 |
| *Leiobunum ventricosum* | JQ432348, JQ432290,  JQ432349, JQ432291, JQ432242,  JQ432350, JQ432292, JQ432243 | USA: TN: Blount Co. | USA: TN: Sevier Co.,  USA: TN: Knox Co.,  USA: KY: Whitley Co. | 4 | 4 | 8 |
| *Leiobunum verrucosum* | JQ432351, JQ432293, JQ432244,  JQ432347, JQ432289, JQ432241 | USA: TN: Cumberland Co. | USA: KY: Whitley Co. | 4 | 5 | 3 |
| *Leiobunum vittatum* | JQ432333, JQ432275, JQ432232,  GQ870651, JQ432334, JQ432276, GQ872155,  JQ432335, JQ432277, JQ432233,  JQ432336, JQ432278, JQ432234,  GQ870652, JQ432337, JQ432279, GQ872156 | USA: TN: Davidson Co. | USA: MO: Carter Co.,  USA: AR: Greene Co., | 5 | 4 | 3 |
| *Leuronychus pacificus* | JQ432368, JQ432310, JQ432253 | USA: AZ: Cochise Co. | USA: AZ: Cochise Co.,  USA: CA: San Diego Co.,  USA: CA: Los Angeles Co.,  USA: CA: Orange Co. | 4 | 3 | 1 |
| *Togwoteeus biceps* | JQ432371, JQ432313 | USA: NM: Taos Co. | USA: NV: White Pine Co.,  USA: NM: Taos Co. | 5 | 2 | 1 |

**References**

1 Hedin M, Derkarabetian S, McCormack M, Richart C, Shultz JW. The phylogenetic utility of the

nuclear protein-coding gene EF-1α for resolving recent divergences in Opiliones, emphasizing intron

evolution. J Arachnol. 2010; 38: 9-20.
